# Serum lncRNA HAND2-AS1 is downregulated in diabetic patients with chronic renal failure and ameliorates cell apoptosis

**DOI:** 10.1186/s13098-020-00548-w

**Published:** 2020-05-07

**Authors:** Changqing Dong, Shengmao Liu, Yanling Li, Yingchun Cui

**Affiliations:** Department of Nephrology, The Second Hospital of Jilin University, No. 218 Ziqiang Street, 130000 Changchun, Jilin People’s Republic of China

**Keywords:** Chronic renal failure, Apoptosis, lncRNA HAND2-AS1

## Abstract

**Background:**

LncRNA HAND2-AS1 has been reported to be a tumor suppressor in several types of malignancy, while its involvement in other human diseases is unclear. Our preliminary RNA-seq analysis revealed the downregulation of lncRNA HAND2-AS1 in diabetic patients with chronic renal failure, indicating the involvement of lncRNA HAND2-AS1 in this disease. This study was therefore carried out to explore the role of lncRNA HAND2-AS1 in the development of chronic renal failure in diabetic patients.

**Methods:**

Mouse podocyte cells and plasma samples of diabetic patients (46 diabetic patients with chronic renal failure, 38 diabetic patients without obvious complications and 42 healthy volunteers) were used in this study. Cell apoptosis assay and PCR were performed.

**Results:**

LncRNA HAND2-AS1 was downregulated in diabetic patients with chronic renal failure but not in diabetic patients without obvious complications. Downregulation of lncRNA HAND2-AS1 distinguished diabetic patients with chronic renal failure from diabetic patients and healthy controls. High glucose environment did not affect the expression of lncRNA HAND2-AS1 in mouse podocyte cells. Overexpression of lncRNA HAND2-AS1 inhibited the apoptosis of mouse podocyte cells under high glucose treatment.

**Conclusions:**

We therefore conclude that lncRNA HAND2-AS1 may participate in the development of chronic renal failure in diabetic patients by regulating cell apoptosis.

## Background

As a major metabolic disorder, diabetes affects almost important organs in the human body [1]. Kidney is the most commonly affected organ in patients with diabetes [2]. Chronic renal failure, which is frequently observed in patients with different types of diabetes, is the major cause of end-stage renal failure or even death in those patients [3]. Even with extensive efforts that have been made on the treatment of diabetic renal diseases, the treatment outcomes are generally poor [4, 5]. Although various signaling pathways have been proved to participate in diabetic injury, pathogenesis of this disease is still unclear [6], leading to difficulties in clinical treatment. Therefore, studies on the molecular mechanism of renal injury in diabetic patients may provide guidance for the treatment.

Besides protein-coding mRNAs, the human genome also contain a large number of non-coding RNAs that participate in both normal physiological and pathological processes [7]. Long non-coding RNAs (lncRNAs), as a subgroup of non-coding RNAs, are key players in human diseases including diabetic complications [8, 9]. LncRNA HAND2-AS1 is a recently identified lncRNA with tumor suppressive functions in several types of human cancer, including osteosarcoma [10], colorectal cancer [11] and endometrioid endometrial carcinoma [12]. Our preliminary RNA-seq data revealed that HAND2-AS1 was downregulated in diabetic patients with chronic renal failure, indicating the involvement of HAND2-AS1 in this disease. We therefore explored the potential function of lncRNA HAND2-AS1 in the development of chronic renal failure in diabetic patients with a focus on cell apoptosis.

## Methods

### Cell lines and serum samples

Mouse podocyte cells were purchased from PrimCells LLC. Podocytes are important for glomerular function. Specifically, podocytes work with mesangial cells to support the function and structure of glomerulus. Podocytes are usually affected in chronic renal failure. We therefore used podocytes to perform in vitro cell experiments. Cell culturing was performed in strict accordance with the manufacturer’s instructions. To assess the effects of high glucose environment on the expression of lncRNA HAND2-AS1, podocytes were cultured in medium containing 5, 10, 20 and 40 mM d-glucose for 6, 12, 24 and 48 h before use.

Serum samples were prepared from blood that was extracted from 46 diabetic patients with chronic renal failure, 38 diabetic patients without obvious complications (such as diabetic retinopathy, diabetic ketoacidosis and diabetic lung, or other severe clinical disorders, such as cancers and heart diseases), and 42 healthy volunteers. The diagnosis of diabetic patients with chronic renal failure was: (1) existence of diabetes; (2) existence of chronic renal failure; (3) excluded chronic renal failure caused by factors unrelated to diabetes. All the participants were admitted by the second hospital of Jilin University from March 2017 and March 2018. Diagnosis was performed according to the standards established by Chinese Medical Association. Patients who received treatments within 3 months before the admission were excluded. The 46 diabetic patients with chronic renal failure included 20 males and 26 females, with an age range of 36–68 years old and a mean age of 52.1 ± 6.8 years old. The 38 diabetic patients included 16 males and 22 females, with an age range of 34–65 years old and a mean age of 50.1 ± 7.2 years old. The 42 healthy volunteers included 20 males and 22 females, with an age range of 35–67 years old and a mean age of 51.3 ± 5.5 years old. This study was approved by the Ethics committee of the second hospital of Jilin University. All participants signed the informed consent. No significant differences in age, gender and BMI were observed among the three groups.

### Real-time quantitative PCR (RT-qPCR)

Total RNAs were extracted using the Monarch^®^ Total RNA Miniprep Kit (NEB). Reverse transcription was performed using the Applied Biosystems™ High-Capacity cDNA Reverse Transcription Kit. SYBR^®^ Green Quantitative RT-qPCR Kit (Sigma-Aldrich) was used to prepare all PCR reaction systems. PCR reaction conditions were: 95 °C for 45 s, followed by 40 cycles of 95 °C for 15 s and 57.5 °C for 38 s. Primers used in PCR reactions were: 5′-GGGTGTTTACGTAGACCAGAACC-3′ (forward) and 5′-CTTCCAAAAGCCTTCTGCCTTAG-3′ (reverse) for human lncRNA HAND2-AS1; 5′-GACCTCTATGCCAACACAGT-3′ (forward) and 5′-AGTACTTGCGCTCAGGAGGA-3′ (reverse) for endogenous control β-actin. Data were processed using 2^−ΔΔCT^ method.

### Cell culture and transfection

HAND2-AS1 expression pEGFPC3 vectors were synthesized by GenePharma (Shanghai, China). HAND2-AS1 siRNA (Catalog # AM16708) and Silencer^®^ Negative Control #1 siRNA (Catalog # AM4611) were purchased from Thermo Fisher Scientific. Transfection was performed using lipofectamine 2000 reagent (11668-019, Invitrogen, Carlsbad, USA) to transfect 15 nM vectors or 40 nM siRNAs into mouse podocyte cells. Transfection with empty vectors or negative control siRNAs was used as negative control (NC) group. Cells without transfection were control cells (C).

### Cell apoptosis assay

Cell apoptosis assay was performed only in cases of the overexpression rate of lncRNA HAND2-AS1 reached 180%. Cells were harvested and single cell suspensions (5 × 10^4^ cells/mL) were prepared. Cell suspensions were transferred to a 6-well plate with 2 mL cell suspension in each well. After that, 5 (control), 10, 20 or 40 mM d-glucose was added and cells were cultured for 24 h. Following digestion with 0.25% trypsin, Annexin V-FITC (Dojindo, Japan) and propidium iodide (PI) staining was performed and apoptotic cells were detected by flow cytometry.

### Statistical analysis

All experiments were performed in triplicate manner and data were expressed as mean ± standard deviation (SD). Data were processed using GraphPad Prim 6 software. Comparisons between 2 groups were performed by Student’s *t* test. Receiver operating characteristic (ROC) curve was used in the diagnostic analysis. Comparisons among multiple groups were performed by one-way ANOVA followed by Tukey test. *P* < 0.05 was considered to be statistically significant.

## Results

### LncRNA HAND2-AS1 was downregulated in serum of diabetic patients with chronic renal failure

**Fig. 1:**
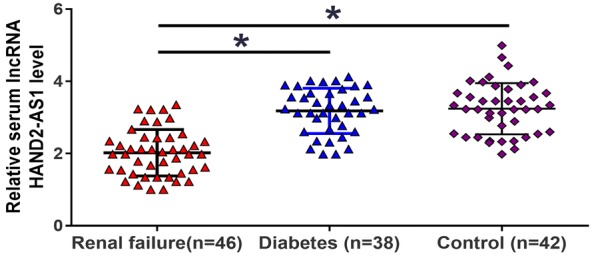
The expression of lncRNA HAND2-AS1 was downregulated in serum of diabetic patients with chronic renal failure. HAND2-AS1 in serum was detected by RT-qPCR. The sample with the lowest expression level was set to “1”, and all other samples were normalized to this sample. Compared with healthy volunteers (Control group), serum levels of lncRNA HAND2-AS1 were significantly reduced in diabetic patients with chronic renal failure (Renal failure group) but not in diabetic patients (Diabetes group) without obvious complications (*, *p* < 0.05)

The qRT-PCR results showed that, compared with diabetic patients (Diabetes group) without obvious complications and healthy volunteers (Control group), the expression levels of lncRNA HAND2-AS1 were significantly lower in the serum of diabetic patients with chronic renal failure (Renal failure group) (Fig. 1, *p* < 0.05). However, no significant differences in serum levels of lncRNA HAND2-AS1 were found between patients without obvious complications and healthy volunteers (Fig. 1, *p* > 0.05).

### Downregulation of serum lncRNA HAND2-AS1 distinguished diabetic patients with chronic renal failure from diabetic patients and healthy controls

**Fig. 2:**
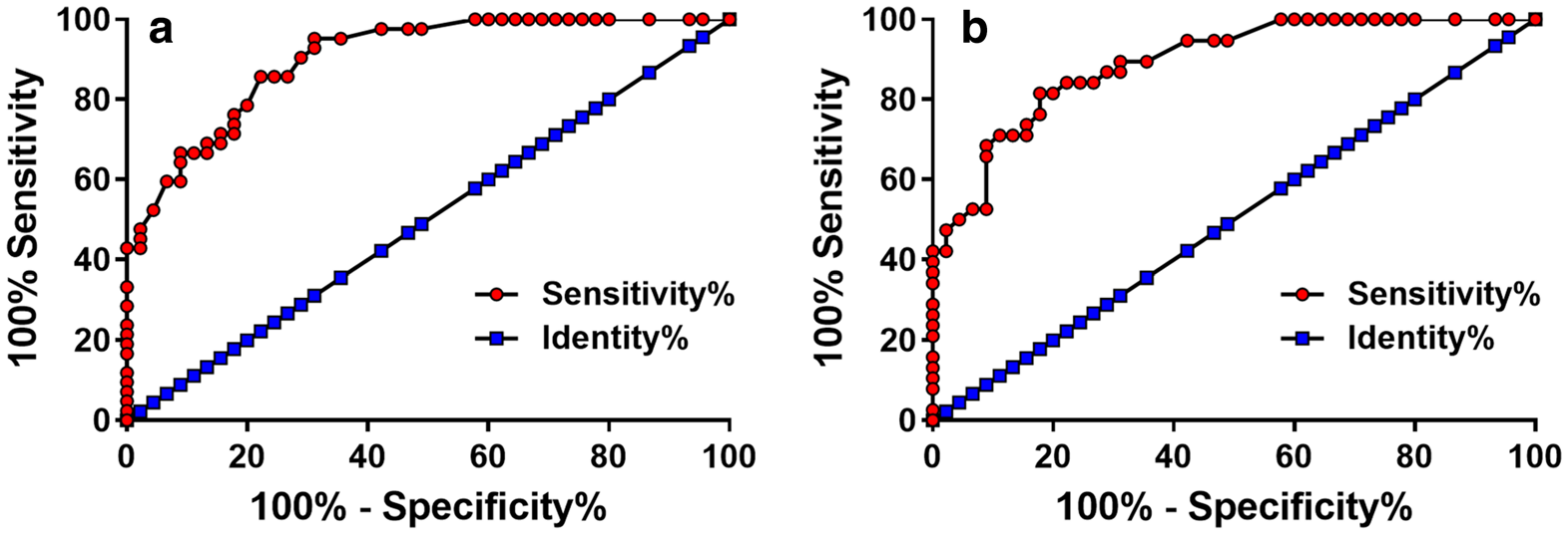
Downregulation of serum lncRNA HAND2-AS1 distinguished diabetic patients with chronic renal failure from diabetic patients and healthy controls. ROC curve analysis was performed with diabetic patients with chronic renal failure as true positive cases and diabetic patients (**a**) or healthy controls (**b**) as true negative cases. ROC curve analysis revealed that downregulation of serum lncRNA HAND2-AS1 distinguished diabetic patients with chronic renal failure from diabetic patients (**a**) and healthy controls (**b**)

ROC curve analysis was performed with patients with chronic renal failure as true positive cases and diabetic patients or healthy controls as true negative cases to evaluate the diagnostic value of serum lncRNA HAND2-AS1 for chronic renal failure in diabetic patients. As shown in Fig. 2, with diabetic patients as reference, area under the curve (AUC) was 0.9024, with standard error of 0.03067 and 95% confidence interval of 0.8423–0.9625 (*p* < 0.0001). With healthy controls as references, AUC was 0.8924, with standard error of 0.03362 and 95% confidence interval of 0.8265–0.9583(*p* < 0.0001).

### High glucose environment did not affect the expression of lncRNA HAND2-AS1 in mouse podocyte cells

**Fig. 3:**
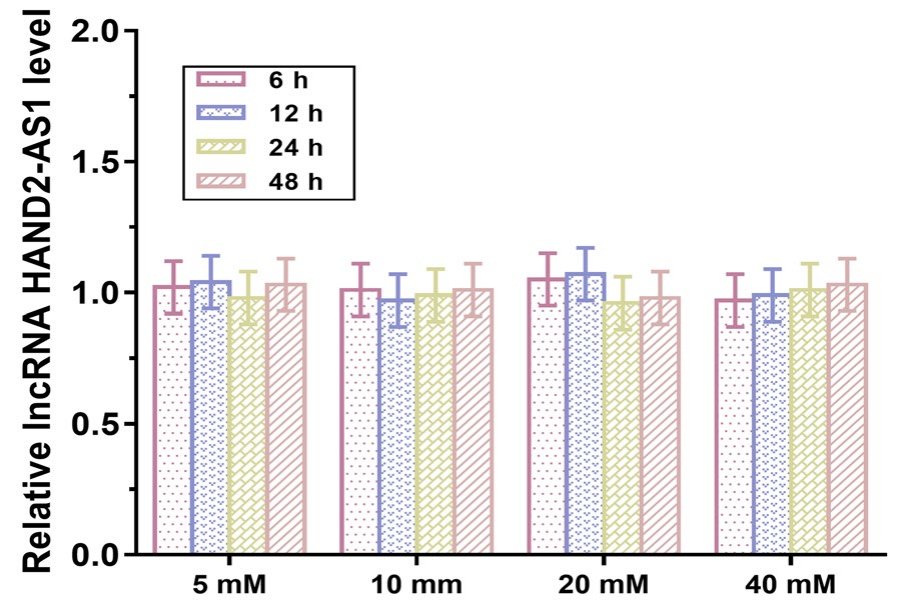
High glucose environment did not affect the expression of lncRNA HAND2-AS1 in mouse podocyte cells. Podocytes were cultured in medium containing 5, 10, 20 and 40 mM d-glucose for 6, 12, 24 and 48 h and the expression of lncRNA HAND2-AS1 was detected by qRT-PCR

To test whether downregulated HAND2-AS1 was induced by high glucose environment, podocytes were cultured in medium containing 5, 10, 20 and 40 mM d-glucose for 6, 12, 24 and 48 h, and the expression of lncRNA HAND2-AS1 was detected by qRT-PCR. As shown in Fig. 3, treatment with different doses of d-glucose for different time periods did not affect the expression of lncRNA HAND2-AS1 in mouse podocyte cells.

### LncRNA HAND2-AS1 inhibited the apoptosis of mouse podocyte cells under high glucose treatment

**Fig. 4:**
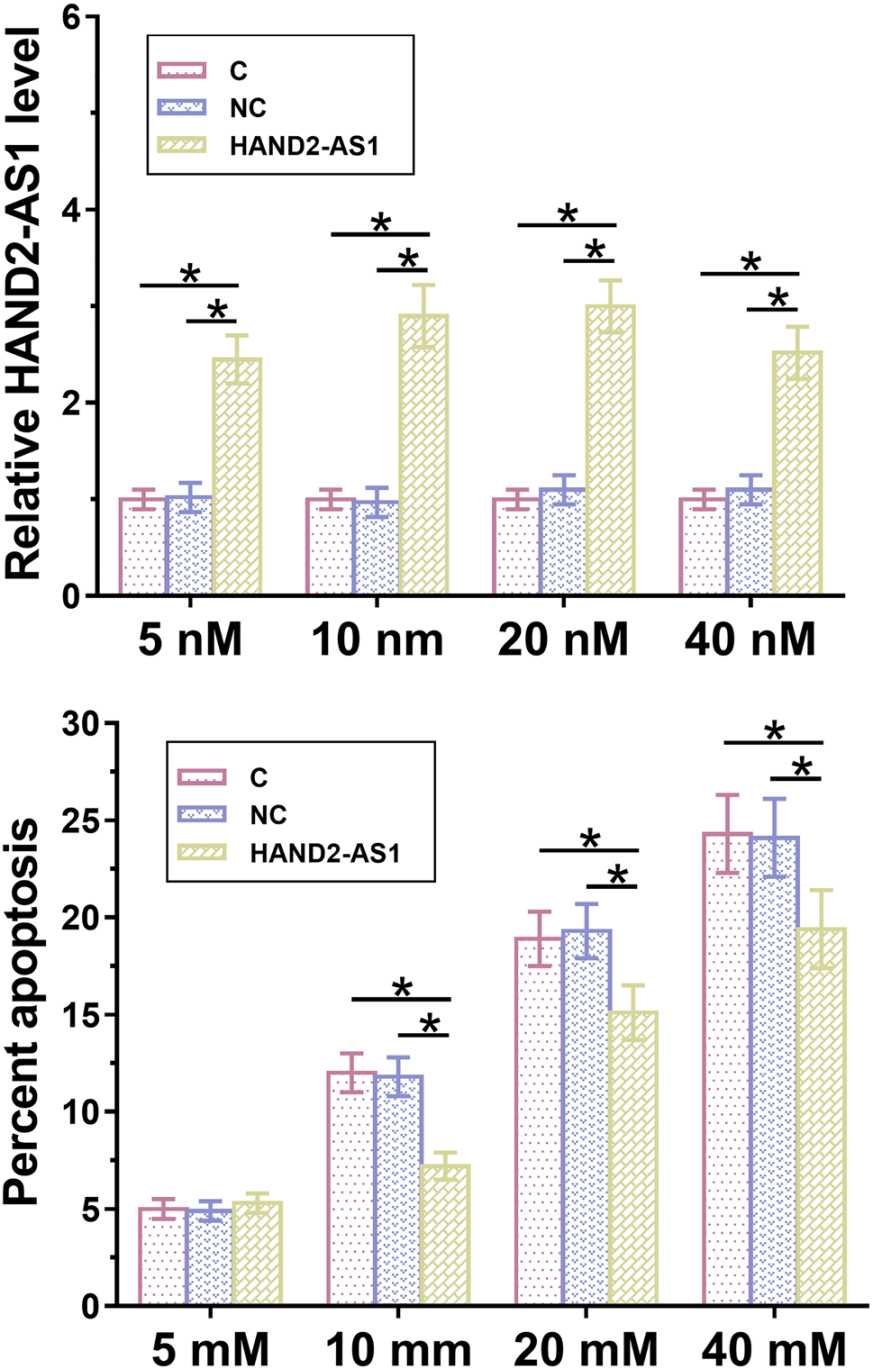
Overexpression of LncRNA HAND2-AS1 inhibited the apoptosis of mouse podocyte cells under high glucose treatment. Apoptosis of mouse podocyte cells under high glucose treatment was detected by performing cell apoptosis assay. Overexpression of HAND2-AS1 inhibited the apoptosis of mouse podocyte cells at each d-glucose concentration except 5 mM, which is within the range of normal blood glucose level (*, *p* < 0.05)

**Fig. 5:**
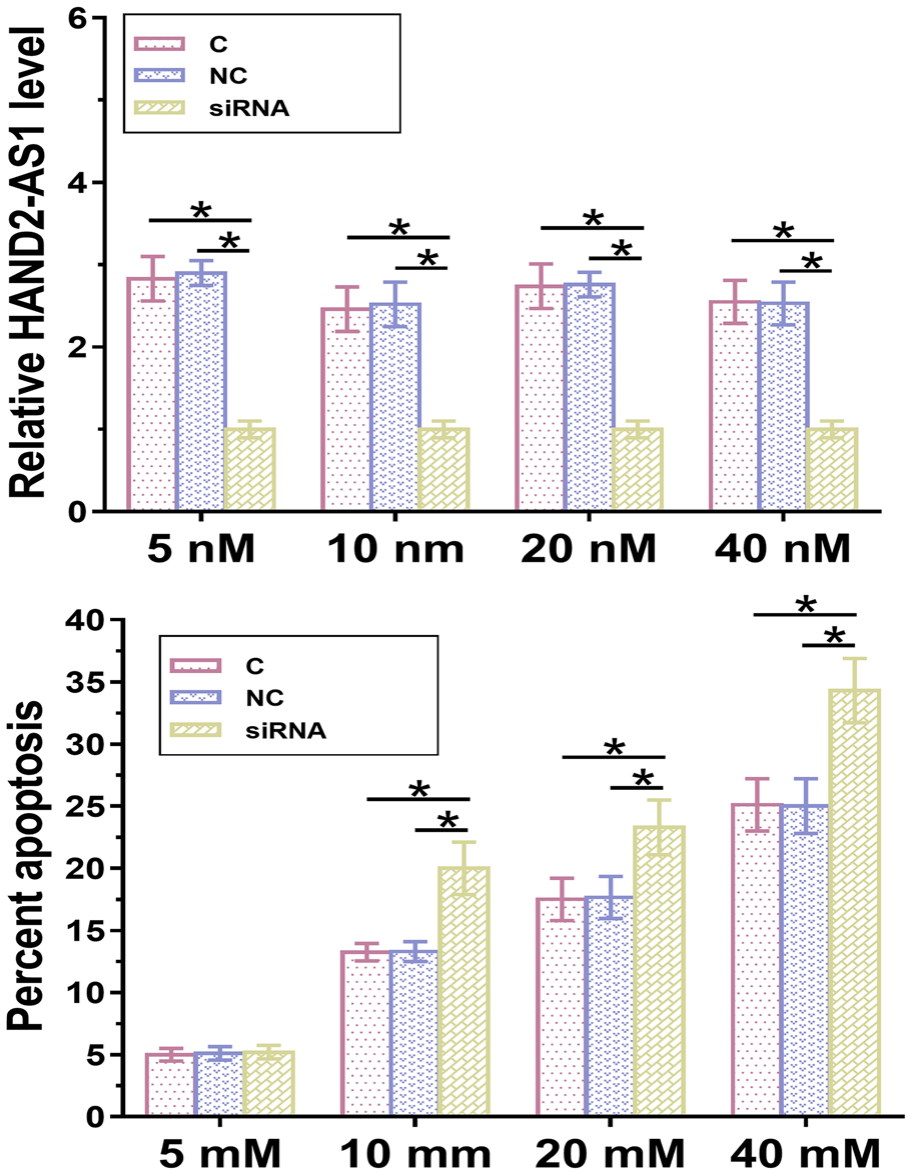
Silencing of lncRNA HAND2-AS1 promoted the apoptosis of mouse podocyte cells under high glucose treatment. Silencing of HAND2-AS1 promoted the apoptosis of mouse podocyte cells at each d-glucose concentration except 5 mM, which is within the range of normal blood glucose level (*, *p* < 0.05)

Podocyte apoptosis contribute to the development or chronic renal failure. Therefore, podocytes were cultured in medium containing 5, 10, 20 and 40 mM d-glucose for 24 h and cell apoptosis was detected by cell apoptosis assay. As showed in Fig. 4, compared with the control group (C) and negative control (NC) group, overexpression of HAND2-AS1 inhibited the apoptosis of mouse podocyte cells at each d-glucose concentration (Fig. 4, *p* < 0.05) except for 5 mM, which is within the range of normal blood glucose level. In contrast, silencing of HAND2-AS1 promoted the apoptosis of mouse podocyte cells (Fig. 5, *p* < 0.05).

## Discussion

LncRNA HAND2-AS1 is a recently identified lncRNA with known functionality in several types of human cancer, including osteosarcoma [10], colorectal cancer [11] and endometrioid endometrial carcinoma [12]. To the best of our knowledge, our study firstly reported the involvement of lncRNA HAND2-AS1 in the development of diabetic chronic renal failure, which is a major type of diabetic complications.

The development of diabetes is accompanied by changes in expression patterns of a large set of lncRNAs [13]. These lncRNAs show upregulated or downregulated expression pattern to promote or inhibit the development of diabetic complications. Onset of chronic renal failure also globally affects the expression of genes including lncRNAs, while the specific functionality of lncRNAs in this disease has rarely been studied [14]. In this study, we observed downregulated expression of lncRNA HAND2-AS1 in diabetic patients with chronic renal failure but not in diabetic patients without obvious complications. Besides, the expression of lncRNA HAND2-AS1 in mouse podocytes was not affect by high glucose treatment. Therefore, lncRNA HAND2-AS1 may specifically participate in renal disease induced by diabetes. In view of the fact that lncRNA HAND2-AS1 was no altered in diabetic patients without obvious complications and high glucose treatment did not affect the expression of lncRNA HAND2-AS1 in mouse podocytes, we speculated that lncRNA HAND2-AS1 might not participate in the initiation of chronic renal failure in diabetic patients. The development of chronic renal failure may cause the downregulation of lncRNA HAND2-AS1 to aggregate the apoptosis of podocytes.

Circulating non-coding RNAs have been widely used in the diagnosis of human diseases [15–17]. In the present study, we detected lncRNA HAND2-AS1 in serum of all participants. ROC curve analysis revealed that downregulation of serum lncRNA HAND2-AS1 distinguished diabetic patients with chronic renal failure from diabetic patients and healthy controls. Therefore, expression of lncRNA HAND2-AS1 in the serum may serve as a promising diagnostic biomarker for chronic renal failure in diabetic patients. However, expression of lncRNA HAND2-AS1 in other diseases is unknown. Therefore, lncRNA HAND2-AS1 should be used with the combination of other biomarkers to improve diagnostic specificity. In addition, more studies are needed to include more clinical disorders, such as other diabetic complications to further test the diagnostic specificity of lncRNA HAND2-AS1. Renal biopsy is not accepted by all patients. So we only used plasma samples in this study. It is known that lncRNAs may be released into blood from the site of synthesis. Our future study will try to identify the site of HAND2-AS1 been synthesized.

Renal cell apoptosis is the major pathological change in diabetic patients with chronic renal failure [18]. Podocytes are differentiated cells in the outer layer of glomerular basement membrane. Podocytes secrete functional molecules to support renal function [19]. In our study, we used mouse podocytes to study the functions of lncRNA HAND2-AS1 in the apoptosis of podocytes under high glucose environment. Our study revealed that overexpression of lncRNA HAND2-AS1 inhibited the apoptosis of podocytes under treatment of different concentrations of d-glucose. Therefore, overexpression of lncRNA HAND2-AS1 may serve as a potential treatment target for diabetic chronic renal failure. Cancer studies have shown that HAND2-AS1 may inhibit cancer cell apoptosis by interacting with apoptosis-related molecules, such as HIF1α [10]. HAND2-AS1 may also interact with those factors to regulate the apoptosis of podocytes. A recent study reported that ASB16-AS1 could interact with the Wnt/β catenin signaling pathway to regulate the apoptosis of lung cancer cells [20]. It is known that the Wnt/β catenin pathway can be activated by high glucose treatment [21]. Therefore, Wnt/β catenin may mediate the role of HAND2-AS1 in the apoptosis of podocytes.

It worth noting that overexpression of HAND2-AS1 did not affect the apoptosis of mesangial cells (data not shown), which are also important for renal functions [21]. High glucose also induced the apoptosis of mesangial cells [22, 23]. Therefore, the inhibitory effect of HAND2-AS1 is likely cell type-specific.

## Conclusions

In conclusion, lncRNA HAND2-AS1 was specifically downregulated in diabetic patients with chronic renal failure. Overexpression of lncRNA HAND2-AS1 may serve as a potential therapeutic target for diabetic chronic renal failure.

## Data Availability

The datasets used and/or analyzed during the current study are available from the corresponding author on reasonable request.

## Abbreviation

lncRNAs: Long non-coding RNAs
